# Advances in Electrochemical Impedance Spectroscopy Detection of Endocrine Disruptors

**DOI:** 10.3390/s20226443

**Published:** 2020-11-11

**Authors:** Lucian-Gabriel Zamfir, Mihaela Puiu, Camelia Bala

**Affiliations:** 1 R&D Center LaborQ, University of Bucharest, 4-12 Regina Elisabeta Blvd., 030018 Bucharest, Romania; lucian-gabriel.zamfir@cdi.unibuc.ro (L.-G.Z.); elenamihaela.puiu@g.unibuc.ro (M.P.); 2 Department of Analytical Chemistry, University of Bucharest, 4-12 Regina Elisabeta Blvd., 030018 Bucharest, Romania

**Keywords:** endocrine, biosensor, impedance, MIP, immunosensor, aptasensor

## Abstract

Endocrine disruptors (EDs) are contaminants that may mimic or interfere with the body’s hormones, hampering the normal functions of the endocrine system in humans and animals. These substances, either natural or man-made, are involved in development, breeding, and immunity, causing a wide range of diseases and disorders. The traditional detection methods such as enzyme linked immunosorbent assay (ELISA) and chromatography are still the golden techniques for EDs detection due to their high sensitivity, robustness, and accuracy. Nevertheless, they have the disadvantage of being expensive and time-consuming, requiring bulky equipment or skilled personnel. On the other hand, early stage detection of EDs on-the-field requires portable devices fulfilling the Affordable, Sensitive, Specific, User-friendly, Rapid and Robust, Equipment free, Deliverable to end users (ASSURED) norms. Electrochemical impedance spectroscopy (EIS)-based sensors can be easily implemented in fully automated, sample-to-answer devices by integrating electrodes in microfluidic chips. The latest achievements on EIS-based sensors are discussed and critically assessed.

## 1. Introduction

Endocrine disruptors (EDs) are environmental contaminants that disrupt the normal functioning of the endocrine system in mollusk, crustacea, fish, reptiles, birds, and mammals. For humans, these compounds may cause cancerous tumors [1,2,3] and infertility [4]. Natural EDs originate in living organisms and can be either hormones (testosterone, estrogen, or progesterone) or mycotoxins such as zearalenone. Synthetic EDs can be found in plastic additives, industrial reagents, and waste. Some of the most common synthetic EDs are precursors in the production of rubber, pesticides and plastic additives such as atrazine, alkylphenols, bisphenol A (BPA) [5], parabens, perfluoroalkyl acids [6], phthalates and polychlorinated biphenyls (PCBs) [7]. A list of relevant EDs is given in Table 1.

A variety of analytical methods have been used for the detection of EDs, including liquid chromatography coupled with mass spectrometry (LC-MS) [8], gas chromatography coupled with mass spectrometry (GS-MS) [9], high-performance liquid chromatography (HPLC) coupled with fluorescence detection [10] or with mass spectrometry [11,12]. These methods usually require laborious and time-consuming steps for sample pre-concentration, and high amounts of reagents. By comparison, electrochemical sensors and biosensors offer advantages such as low cost, portability, and do not require complex pretreatment steps. Moreover, biosensors can be used for selective, fast, and direct detection of analytes in real samples.

**Table 1:** List of relevant EDs compounds.

Analyte	IUPAC Name	Chemical Structure	Molecular Weight (g/mol)	Source	Ref.
17β-estradiol (E2)	(8R,9S,13S,14S,17S)-13-Methyl-6,7,8,9,11,12,14,15,16,17-decahydrocyclopenta[a]phenanthrene-3,17-diol	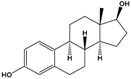	272.388	endogenous hormone, medication	[13]
Acetamiprid (AAP)	N-[(6-chloro-3-pyridyl)methyl]-N′-cyano-N-methyl-acetamidine	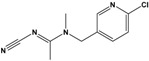	222.678	insecticide	[14]
Atrazine (ATZ)	6-chloro-N2-ethyl-N4-(propan-2-yl)-1,3,5-triazine-2,4-diamine	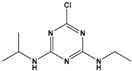	215.69	herbicide for grassy weeds in crops	[15]
Pentabromodiphenyl ether (BDE-47)	2,2′,4,4′-Tetrabromodiphenyl ether	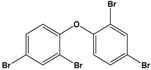	485.79	flame retardant	[16]
Bisphenol A (BPA)	4,4′-(propane-2,2-diyl)diphenol	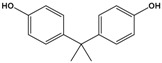	228.291	precursor to polycarbonates, plastic and epoxy resins	[17]
Carbendazim (CBZ)	methyl 1H-benzimidazol-2-ylcarbamate	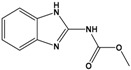	191.187	fungicide	[18]
Cortisol	11β,17α,21-Trihydroxypregn-4-ene-3,20-dione	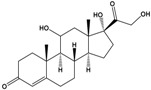	362.46	endogenous hormone, medication	[19]
Dibutyl phthalate (DBP)	Dibutyl benzene-1,2-dicarboxylate	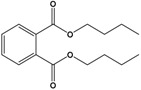	278.348	plasticizer	[20]
Dichloro-diphenyl-trichloroethane (DDT)	1-chloro-4-[2,2,2-trichloro-1-(4-chlorophenyl)ethyl]benzene	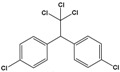	354.48	pesticide	[21]
Di(2-ethylhexyl) phthalate (DEHP)	Bis(2-ethylhexyl) benzene-1,2-dicarboxylate	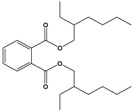	390.564	plasticizer	[22]
Microcystin-LR (MC-LR)	(5R,8S,11R,12S,15S,18S,19S,22R)-15-[3-(diaminomethylideneamino)propyl]-18-[(1E,3E,5S,6S)-6-Methoxy-3,5-dimethyl-7-phenylhepta-1,3-dienyl]-1,5,12,19-tetramethyl-2-methylidene-8-(2-methylpropyl)-3,6,9,13,16,20,25-heptaoxo-1,4,7,10,14,17,21-heptazacyclopentacosane-11,22-dicarboxylic acid	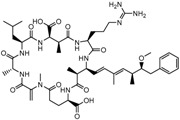	995.189	cyanobacteria toxin	[23]
Norfluoxetine (NorFLX)	(S)-3-Phenyl-3-[4-(trifluoromethyl)phenoxy]propan-1-amine	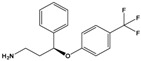	295.305	antidepressant	[24]
3,3’,4,4’-tetrachlorobiphenyl (PCB-77)	3,3′,4,4′-tetrachloro-1,1′-biphenyl	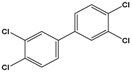	291.99	flame retardants, plasticizers, dielectric and heat transfer fluids	[25]
Testosterone	(8R,9S,10R,13S,14S,17S)-17-Hydroxy-10,13-dimethyl-1,2,6,7,8,9,11,12,14,15,16,17-dodecahydrocyclopenta[a]phenanthren-3-one	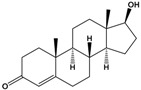	288.431	endogenous hormone, anabolic steroid	[26]
Tributyltin hydride	tributylstannane	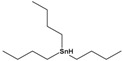	291.06	precursor in organic synthesis	[27]
Zearalenone (ZEN)	(3S,11E)-14,16-Dihydroxy-3-methyl-3,4,5,6,9,10-hexahydro-1H-2-benzoxacyclotetradecine-1,7(8H)-dione	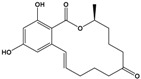	318.369	mycotoxin	[28]

Electrochemical impedance spectroscopy (EIS) is a sensitive technique which can be used to monitor biomolecular events occurring at the electrode surface. These events include affinity interactions involving peptides, receptors, nucleic acids, whole cells, and antibodies.

This work reviews recent trends in the newly developed EIS sensors for the detection of EDs using different modified surfaces and various bioreceptors. In this review, significant examples of impedimetric sensors and biosensors for the detection of EDs are discussed and critically reviewed.

## 2. Basic Elements of EIS-Based Sensors

### 2.1. Principle of EIS Detection

Electrochemical impedance spectroscopy (EIS) is an electrochemical technique that measures the impedance properties of an electrochemical system using a large range of frequencies. Here, the electrochemical process is described by an electrical circuit consisting of resistance, capacitors, and constant phase elements combined in parallel or in series.

The most widely used model for describing processes at the electrochemical interface is the Randles equivalent circuit [29], consisting of electrolyte resistance (R_s_), charge-transfer resistance (R_ct_) at the electrode/electrolyte interface, double-layer capacitance (C_dl_), mass transfer resistance (R_mt_) and Warburg impedance (W). The R_s_ parameter is determined by the conductivity of the solution and the distance between the electrodes. The double layer capacitance depends on the electrode area, nature, and electrolyte’s ionic strength. R_ct_ and W represent the Faradaic impedance. R_ct_ depends on the charge transfer kinetics and can be thought of as the ratio of overpotential to current in the absence of mass transfer limitation. The linear segment registered at low frequencies is attributed to the Warburg diffusion element, the impedance being controlled by the diffusion process in this region.

Equivalent circuit models can serve to describe the electrochemical, chemical, and physical processes occurring at the electrode surface, since each circuit component can be assigned to a physical process in the electrochemical cell. Electrochemical reactions involve electrolyte resistance, adsorption of electroactive species, charge transfer at the electrode surface, and mass transfer from the bulk solution to the working electrode surface. Each electrochemical process is represented by an electrical circuit that consists of capacitors, resistance and constant phase elements that are connected in parallel or in series.

EIS has been proven to be a useful tool for the analysis of interfacial or bulk electrical properties of the electrode, which can be used to quantitatively determine electrochemical processes [30]. EIS enables label-free detection with high signal-to noise ratio amenable to on-site analysis.

### 2.2. Types of Impedance Sensors

**Figure 1:**
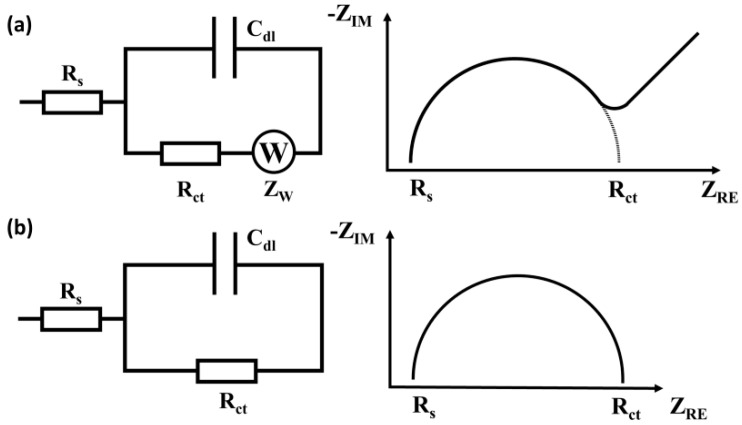
Common equivalent circuit models for EIS biosensors. (**a**) Faradaic systems with the Nyquist plot. (**b**) Non-Faradaic systems with the Nyquist plot. R_s_ = solution resistance; R_ct_ = charge transfer resistance; C_dl_ = double layer capacitance; W = Warburg element; Z_RE_ = real impedance component; Z_IM_ = imaginary impedance component.

Impedance sensors can be classified according to the relation between the charge transfer process and the parameters measured into Faradaic or capacitive sensors (Figure 1).

(A) Faradaic impedance sensors use electrodes with conductive surfaces; the measurements require redox-active molecules in solution, such as hexacyanoferrate(II)/(III) anions or hexaammineruthenium (II)/(III) cations [31]. Charge transfer resistance (R_ct_) is the main parameter that characterizes the electrochemical process at the sensor’s surface. The surface-binding of non-conductive molecules blocks the electron transfer (ET), causing an increase in R_ct_. Conversely, the binding of conductive molecules or molecules able to catalyze redox reactions leads to the decrease in R_ct_.

(B) Non-Faradaic (Capacitive) sensors are systems where the sensing surface is covered by an insulating layer. The double-layer capacitance (C_dl_) is the main parameter that characterizes the reactions occurring at the electrolyte/electrode interface [32]. The binding of the molecules to the surface usually decreases the value of C_dl_.

### 2.3. Electrochemical Impedance Spectroscopy for Biosensing Applications

EIS also presents the possibility of carrying out label-free experiments, unlike other electrochemical techniques, such as amperometry and voltammetry [30]. The EIS sensors are often based on various modified surfaces that increase the amount of bioreceptor on the surface, and consequently the performance of the biosensor.

A biosensor is an analytical device in which a biological component, called a bioreceptor, is integrated or in direct contact with a physicochemical detector that turns the biological signal into a measurable analytical signal [33]. Biosensors offer a simple, rapid and cost-effective alternative for the detection of harmful compounds [34]. The bioreceptor is the component that interacts in a specific manner with the analyte, and can be an enzyme, antibody, nucleic acid, organelle, cell, or an organic tissue. The selectivity of the biosensor is determined by the affinity features of the biological receptor. The signal generated by the interaction between the analyte of interest and the biological recognition element is then transformed by a transducer to an optical or electrical readout. The EIS technique has been used to monitor specific interactions occurring at the electrode surface between a receptor and the specific target analyte; the impedance is used to quantitatively assess the analyte. The advantages of EIS biosensors are their sensitivity, simplicity, and possibility to achieve real-time detection. The use of EIS also has several disadvantages such as being sensitive to the surrounding environment, often requiring a Faraday cage to reduce noise, bulky experimental setups and the need for theoretical simulation for data analysis [35].

## 3. EIS Sensors for EDs Detection

Recently reported EIS sensors and biosensors for EDs detection have used various metal oxides [36], metal organic frameworks (MOFs) [37] and molecularly-imprinted polymers (MIPs) [38], either as supporting layers, or recognition elements. The immobilization strategy and the detection principles will be discussed further.

### 3.1. Molecular-Imprinted Polymer Sensors

MIPs are artificial recognition elements used in the development of sensors due to their high selectivity, chemical and thermal stability, and easy customization compared to receptors from biological sources. MIPs are created by polymerizing a functional monomer in the presence of the analyte template [39]. After the removal of the template, cavities with specific shapes are formed, allowing a highly selective interaction with the target analyte [40]. MIPs bind to the target molecules, leading to variations in physical parameters at the sensor surface, such as mass, absorbance, or electron transfer (ET) rate. In the case of electrochemical sensors, the specific interaction often hampers the electron transfer between the electrode and the redox probe in the solution, which can be quantified through EIS measurements. MIPs have been designed for the extraction of various EDs, such as 17β-estradiol [38] or bisphenol A (BPA) [41] from contaminated environments. In MIP-based EIS sensors, the ED molecules fill the MIP cavities, hindering ET and thus increasing the R_ct_ value. MIPs are usually immobilized on the sensor surface and interact with the analyte in solution. 

**Figure 2:**
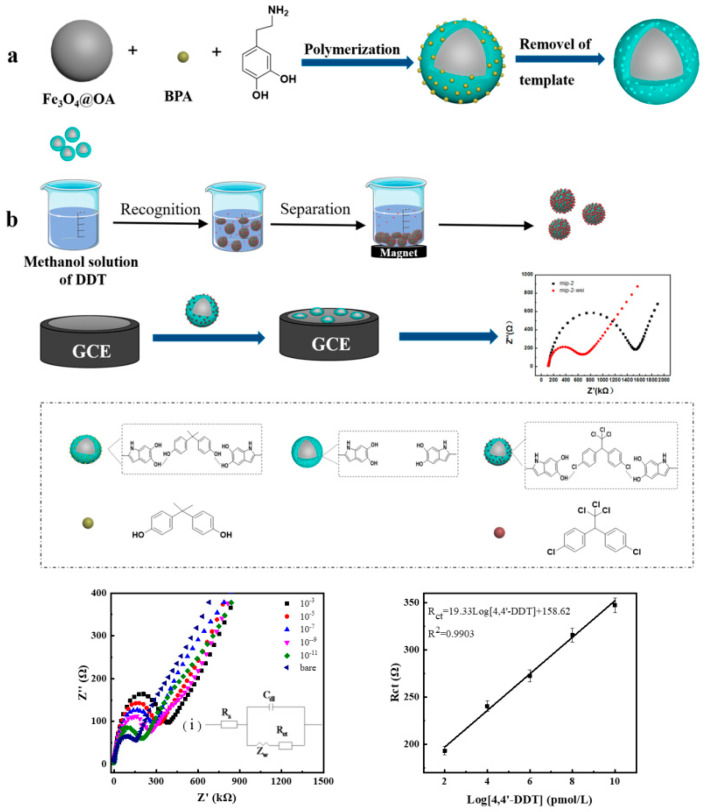
Schematic illustration of (**a**) synthesis of PDA@Fe_3_O_4_-MIP MNPs and (**b**) stepwise preparation process of the electrochemical impedance sensor for 4,4′-DDT detection. Reproduced from [42] with permission of Elsevier.

A recent MIP-based sensor was developed for the impedimetric detection of 4,4′-dichlorodiphenyltri-chloroethane (4,4′-DDT) by Miao et al. [42], based on magnetic Fe_3_O_4_ and polydopamine, using BPA as virtual template and dopamine as functional monomer. The obtained polydopamine (PDA)@Fe_3_O_4_-MIP magnetic nanoparticles (MNPs) were incubated in the sample solution containing 4,4′-DDT; the composite material containing 4,4′-DDT molecules was separated from the solution with the help of a magnet and deposited further on a glassy carbon electrode (GCE) (Figure 2). The impedance of the resulting material was measured using EIS on a GCE. The method was used to determine 4,4′-DDT concentration over a range from 1 × 10^−11^ to 1 × 10^−3^ M with a limit of detection (LOD) of 6 × 10^−12^ M.

Radi et al. [43] developed a MIP sensor for the detection of zearalenone (ZEN) based on o-phenylenediamine (o-PD) electropolymerized on a screen-printed gold electrode and using the ZEN molecule as the template. The sensor showed low LOD and low cross-reactivity with other *Fusarium* mycotoxins. The latest achievements in the field are summarized in Table 2. 

MIPs can also be prepared using biomolecules such as DNA fragments as templates. A MIP sensor developed by Ensafi et al. [52] using a DNA-based MIP achieved very low LODs for BPA. The modified sensor surface was obtained by electrodepositing AuNPs on a glassy carbon electrode and depositing a thiolated DNA sequence with high affinity for BPA (p-63) and free BPA. Pyrrole was electropolymerized on the surface of the GCE to entrap the BPA@p-63 complex, obtaining the MIP cavities that act as binding sites for BPA. The obtained PPY/@p-63/AuNP/GCE achieved one of the lowest LOD reported for an electrochemical sensor for BPA (80 aM). AuNPs enhance the active surface area of the electrode and, thus, the density of active sites, ultimately leading to improved sensitivity.

### 3.2. Metal Composite-Based Sensors

Metal composites represent a combination of two metals or a combination between a metal and another type of material, such as a polymer. Metal–polymer composites have a large surface area and enhanced electrical conductivity due to their mesoporous structures. Metal–organic frameworks (MOFs) are a class of compounds consisting of metal ions or clusters coordinated to organic ligands to form one-, two-, or three-dimensional structures. Due to their customizable structure and functionality, high porosity and large internal surface area, MOFs have great potential in electrochemical sensing applications [53]. However, there are few notable works reporting MOF-based sensors for EDs s detection with moderate performance, most of them not being tested on real samples.

**Figure 3:**
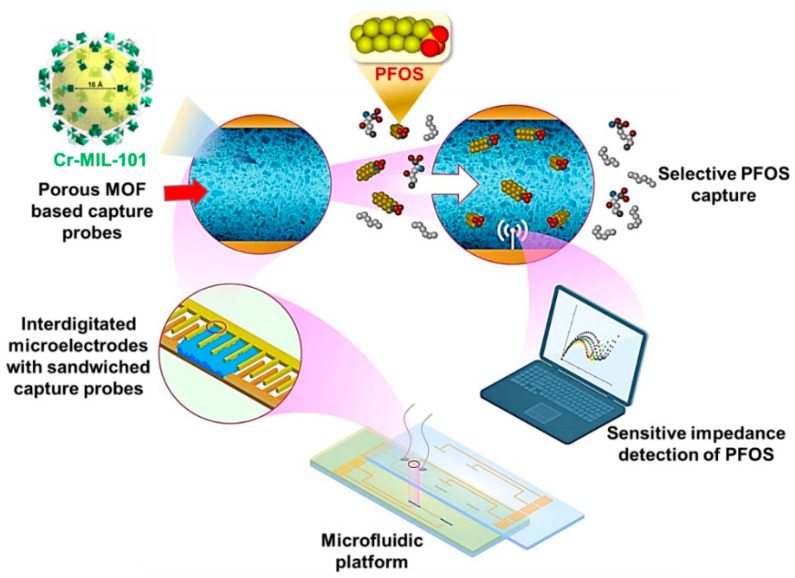
Schematic representation of PFOS detection using nanoporous MOF-based receptors immobilized on capacitive electrodes. The modified interdigitated microelectrodes were integrated within a microfluidic flow-through platform. Reproduced from [37]. Copyright (2020) American Chemical Society.

A simple approach is based on the immobilization MnO_2_ on a gold electrode and this was integrated into a microfluidic platform [36]. The system was used for the detection of BPA within a linear range of 1 nM–62.5 µM with a detection limit of 0.66 µM. Cheng et al. [37] prepared a novel microfluidic system for the detection of the perfluorooctane sulfonate (PFOS), perfluoroalkyl pollutant and ED. The modified sensor uses a non-conductive mesoporous chromium terephthalate metal–organic framework (Cr-MIL-101, MIL Matérial Institut Lavoisier) as MOF receptor. The Cr-MIL-101 provided a higher surface area and higher affinity towards PFOS. The Cr-MIL-101 was immobilized on an interdigitated microelectrode array with sandwiched capture probes (Figure 3). The modified electrode was adapted into a microfluidic lab-on-a-chip sensing platform that achieved the detection of PFOS within a linear range of 0.5 ng/L–50 µg/L with a detection limit of 0.5 ng/L, lower than other non-electrochemical detection methods.

### 3.3. Graphene, Carbon-Nanotubes and Cyclodextrins Based Sensors

Graphene is an allotrope of carbon with 2D layers of sp^2^-hybridized carbon. It is used for sensor modification due to its high electric conductivity and large surface area, which is amenable to functionalization with biomolecules. Single carbon nanotubes (SWCNT) and multiwalled carbon nanotubes (MWCNT) were used to modify the electrochemical transducers, due to their high electron transfer rate, surface area, minimization of the surface fouling and stability [54].

A simple approach for detecting polychlorinated biphenyls such as PCB-77 was developed by Wei et al. [55], where pyrenecyclodextrin (PyCD) was immobilized a SWCNT-modified GCE. The presence of the pyrenyl group on the CD favored the attachment on the surface of the carbon nanotubes trough π–π stacking. The PCB-77 molecules formed complexes with the immobilized PyCD that hindered ET between the ferro/ferricyanide anions and the sensor surface. Thus, the R_ct_ parameters increased with the concentration of PCB-77. The most relevant works on graphene, nanotubes and cyclodextrin modified EIS sensors are summarized in Table 3.

Recently, Hsine et al. [56] combined the use of a porphyrin derivative with that of thermally reduced graphene oxide (TRGO), which also can be attached using π–π interactions. BPA molecules were absorbed on the surface of the nanocomposite, increasing the membrane resistance, which was quantified with EIS.

## 4. EIS Biosensors for the Detection of EDs

### 4.1. Immunosensors

Immunoassays are based on the specific interaction between an antigen and the corresponding antibody (Ab), which can be transduced into a measurable physical signal [59]. Immunosensors can be prepared using monoclonal, polyclonal or recombinant Abs. Immunosensors have been used for the detection of EDs, such as DES, estradiol, phthalates and bisphenol A [60]. The bonds between antibodies and antigens are relatively weak and can be dissociated by changing the properties of the environments, i.e., pH and ionic strength. Singh et al. developed a simple label-free immunosensor for 17β-estradiol [61]. Silver wire electrodes were modified with an 11-mercaptoundecanoic acid self-assembled monolayer (SAM) and the 1-ethyl-3-(3-dimethylaminopropyl)carbodiimide (EDC)-N-hydroxy succinimide (NHS) chemistry was used to covalently bind the 17β-estradiol monoclonal antibodies. In this case, the parameter measured was the capacitance.

Another interesting approach was reported by Supraja et al. [62]. They have used MWCNT-ZnO hybrid nanofibers for electrode modification. MWCNT-ZnO nanofibers were deposed on GCE and treated with 3-sulfanylpropionoic acid to ensure the presence of -COOH groups. Anti-atrazine antibodies were immobilized via EDC-NHS coupling, and the remaining sites were blocked with BSA. The use of the MWCNT-ZnO nanofibers enhanced the surface area by 33% compared to the electrodes modified only with ZnO, and thus increased the amount of anti-atrazine antibodies on the working electrode. Wang et al. [43] developed an immunosensor for the mycotoxin ZEN based on enzyme-catalyzed precipitation. Strip-shaped Co_3_O_4_ (ssCo_3_O_4_), a material with peroxidase-like activity, catalyzes the oxidation of 4-chloro-1-naphthol. The insoluble precipitate generated in this reaction was nonconductive and accumulated onto electrode, hampering the electron transfer between the redox probe to the working electrode. The glassy carbon electrode was first modified with hyaluronic acid (HA)-functionalized TiO_2_ mesocrystals (TiO_2_ MCs). Then, an antibody, Ab_1_, was covalently attached to HA via EDC-NHS coupling. In this sandwich assay, the ZEN molecules bind to the Ab_1_/HA-TiO_2_ MC/GCE immunosensor. Ab_2_@ssCo_3_O_4_ conjugate binds the captured ZEN molecules, causing the accumulation of insoluble products at the surface and the increase in R_ct_; thus, the concentration of ZEN was determined through EIS measurements. The most recent EIS immunosensors for ED detection are presented in Table 4.

Chen et al. used a tyramine-modified rutile TiO_2_ mesocrystals (Tyr-RMC) to label a ZEN mimic peptide [63]. The peptide@Tyr-RMC conjugate binds an antibody-modified GCE in competition with free ZEN. Although the peptide-based sensor was designed for an assay based on dual-signal readout competitive enzyme-linked immunosorbent assay (C-ELISA), the ZEN could also be detected by EIS, with the signal gradually decreasing while the concentration increases.

### 4.2. Aptamer-Based Biosensors

Aptamers are oligonucleotides that bind to a specific target. Because of their in vitro selection and production, the relatively new technology of aptamers has emerged as an alternative to antibodies, as they are obtained through chemical synthesis, with high reproducibility, and their production is not dependent on living organisms. They can be easily regenerated, have a much longer shelf life, and can be stored at ambient temperature. Several significant impedimetric aptasensors for the detection of EDs are presented in Table 5.

**Figure 4:**
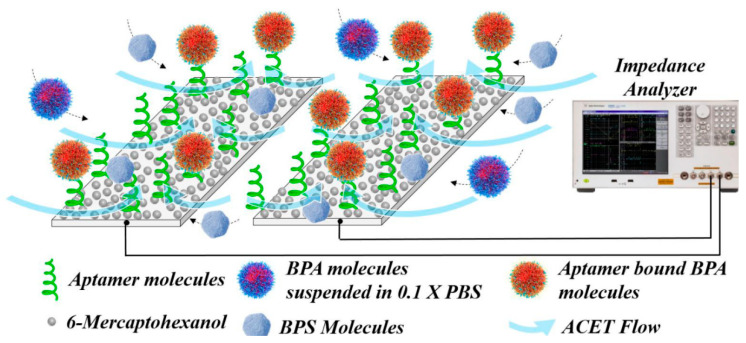
Excitation of ACET effect is integrated into capacitance measurement during a BPA detection experiment. The ACET flow carries BPA particles, along with Bisphenol S and Bisphenol F as interfering compounds, toward functionalized sites and accelerate the sensor response. Reproduced from [66] with permission of Elsevier.

Kang et al. [64] developed a microfluidic aptasensor for the detection of BPA using an anodized aluminum oxide-based capacitive sensor. A gold electrode surface was immobilized on top of the Anodized aluminum oxide (AOO) surface and this allowed the immobilization of a thiol-modified BPA aptamer. The capacitance of the system decreased due to the conformational change of immobilized aptamer at the binding to the BPA molecules. The sensing surface was encased in a microfluidic channel and this allowed a real-time capacitance measurement during the binding of BPA to the immobilized aptamer. A capacitive aptamer microelectrode array has also been used for the detection of BPA, with aptamers immobilized on an array made of interdigitated aluminum microelectrodes [65]. The method combines AC electro-kinetics (ACEK) effects and capacitance measurement. The main advantages of the detection system are the low cost of the disposable microelectrodes, fast response time (20 s), and the limit of detection reported for BPA is 2.8 fg/mL. Mirzajani et al. developed a BPA aptasensor in an ACET (alternating current electrothermal) flow based system [66]. The BPA aptasensor is based on printed circuit board technique combined with capacitive sensing (Figure 4). The ACET effect generates directional microflows and long-range convection of the BPA molecules to the electrode surface. To selectivity of the biosensors is also validated by using a flow containing a mixture of BPA molecules along with the interfering compounds bisphenol S and bisphenol F. The capacitive biosensor achieved one of the lowest LODs for BPA reported in the literature (152.93 aM).

**Table 4:** Impedimetric immunosensors for the detection of EDs.

Analyte	Platform	Linear Range	LOD	Real Sample	Advantages	Limitations	Ref.
E2	mAb/11-MUA/Ag wire electrode	1–200 pg/mL	1 pg/mL	water	Fast response time; Label-free; Low sample volume; High sensitivity	Low stability compared to MIP and aptamer-based systems; Risk of cross-reactivity	[61]
E2	Ag-ZnONRs-16-PHA-mAb-E2	0.1–200 pg/mL	0.1 pg/mL	tap and packaged water	Label-free; Low sample volume; High sensitivity; Fast response time	Multiple preparation and optimization steps	[67]
ATZ	GCE/MNF/MPA/EDC-NHS/Ab	1 zg/mL–1 μg/mL	0.22 zg/mL	water	Label-free; High sensitivity; Simple protocol; Wide linear range	Requires blocking of non-specific sites	[68]
ATZ	Ab-SPA-MWCNT-ZnO/GCE	10 zM–1 µM	5.368 zM	-	Label-free; Low detection limit; Wide linear range	Multiple preparation steps; Requires blocking of non-specific sites	[62]
BDE-47	Ab/11-MUA/Au electrode	0.01–0.40 μg/mL	1.3 ng/mL	-	Facile antibody regeneration	Narrow linear ranges; Risk of non-specific binding	[69]
NorFLX		0.02–0.32 μg/mL	8.5 ng/mL	-			[69]
BPA	Ab-nano-CP/GCE	1–100 ng/mL	0.3 ± 0.07 ng/mL	human serum	Label-free	Narrow linear ranges	[70]
Cortisol	Ab/β-MnO_2_ CNs/GCE	0.1 pM–1500 pM	0.023 pM	human sweat and saliva	High stability; Wide linear range	Requires blocking of non-specific sites; Requires sample deoxygenation	[71]
DBP	antigen/CS/MWCNTs@GONRs/GCE; Ab2-AuNP conjugate	5–500 ng/L	7 ng/mL	pure, tap, pond and river water	Low detection limit; Low sample volume	Risk of non-specific binding	[72]
MC-LR	Ab/MC-LR/3D GF electrode	0.05–20 μg/L	0.05 μg/L	tap water	High sensitivity; Low detection limit	Additional preparation steps for the electrode materials; Low stability of bound antibodies	[73]
ZEN	Ab_2_@ssCo_3_O_4_/ZEN/Ab1/HA-TiO_2_ MC/GCE	0.1 fg/mL–10 pg/mL	33 ag/mL	beer	Low detection limit; Use of enzyme mimic	Low resolution of sensing system; Requires redox label	[74]
ZEN	peptide@Tyr-RMC, Ab/poly(Gly)/AuNCs/CNHs/GCE	10^−6^–10 ng/mL	10^−6^ ng/mL	soybean sauce	Fast response time; Wide linear range	Multiple preparation and optimization steps; Requires blocking of non-specific sites; Requires redox label	[63]

11-mercaptoundecanoic acid: 11-MUA; Au nanocones: AuNCs, 16-phosphonohexadecanoic acid 16-PHA; 2,2′,4,4′-Tetrabromodiphenyl ether: BDE-47; carbon nanohorns: CNHs; dithiobis-N-succinimidyl propionate: DTSP; hyaluronic acid: HA; monoclonal antibody: mAb; monoethanolamine: MEA; electrospun manganese oxide nanofibers: MNF; manganese oxide cacti-like nanostructures: β-MnO2 CNs; 3-mercaptopropionic acid: MPA; nano-particle comprised conducting polymer: nano-CP; polyglycine: poly(Gly); Zinc Oxide nanorods: ZnONRs.

**Table 5:** Impedimetric aptamer (Apt) and DNA biosensors for the detection of EDs.

Analyte	Platform	Linear Range	LOD	Real Samples	Advantages	Limitations	Ref.
**E2**	Apt/dendritic Au/BDD electrode	1 × 10^−14^–1 × 10^−9^ M	5 × 10^−15^ M	river water	High sensitivity; High specificity	-	[75]
**E2**	Apt/CDs/SPCE	1.0 × 10^−7^–1.0 × 10^−12^ M	0.5 × 10^−12^ M	river water	High selectivity; High stability	Additional preparation steps for the electrode materials	[76]
**AAP**	MCH/Apt/AuNPs/Au electrode	5–600 nM	1 nM	wastewater, tomatoes	Simple modification protocol	Relatively low sensitivity; Relatively narrow linear range	[77]
**AAP**	MCH/Apt/Au/MWCNT-rGONR/GCE	5 × 10^−14^–1 × 10^−5^ M	1.7 × 10^−14^ M	-	Wide linear range	Long preparation procedure	[78]
**AAP**	MCH/Apt/GOPTS/PtNPs/PMMA/IDE	10 pM–100 nM	1 pM	tap and bottled mineral water	High sensitivity	Long incubation time (60min) due to large custom-made electrochemical cell	[79]
**ATZ**		100 pM–1 μM	10 pM				[79]
**BPA**	Apt-Au/AOO, capacitive biosensor	1 × 10^−9^–1 × 10^−7^ M	100 pM	-	High sensitivity; Microfluidic system	Requires custom-made electrodes; Single-use device	[64]
**BPA**	Apt/Cu^2+^/PPY-NTA/GCE	10^−11^–10^−6^ M	1.24 × 10^−12^ M	-	Simple modification protocol; Wide linear range	-	[80]
**BPA**	MCH/Apt/Au-NPs/BDD	1 × 10^−14^–1 × 10^−9^ M	7.2 × 10^−15^ M	spiked milk	Low detection limit; Simple modification protocol		[81]
**BPA**	Apt/interdigitated aluminum microelectrode, capacitive biosensor	1 fM–1 pM	10 fM	human serum	Fast response time (20s); High sensitivity; Low sample volume	-	[65]
**BPA**	PPY/BPA@p-63/AuNP/GCE	0.5 fM–5 pM	80 aM	fresh milk, milkpowder, tap and pretreated water in baby glass	Low detection limit; Short assay time	-	[52]
**BPA**	Apt/IDE, capacitive biosensor	1 fM–10 pM	152.93 aM	-	Fast response time (20s); Low detection limit	-	[66]
**BPA**	MB-DNA/MWCNTs-CS/PdNPs/C60/GCE	0.5–25 μM	0.35 μM	-	Detection of DNA damage induced by ED	Relatively low sensitivity; Narrow linear range	[82]
**CBZ**	MCH/Apt/Au electrode	10 pg/mL–10 ng/mL	8.2 pg/mL	mango juice, soya milk, tomato, plum	Simple modification protocol	Long preparation time	[83]
**CBZ**	MCH/Apt/AuNPs/1-AP-CNHs/GCE	1–1000 pg/mL	0.5 pg/mL	lettuce andorange juice	High selectivity	Long preparation time	[84]
**DEHP**	Apt/AuNPs/MCH/Au	7.629 pg/mL–2 µg/mL	0.103 pg/mL	tap water	High sensitivity; Low detection limit	-	[85]

1-aminopyrene modified carbon nanohorns: 1-AP-CNHs; anodized aluminum oxide: AOO; boron-doped diamond: BDD; fullerene C60: C60; carbon dots: CDs; chitosan: CS; (3-glycidyloxypropyl) triethoxysilan: GOPTS; interdigitated electrode: IDE; methylene blue: MB; 6-mercapto-1-hexanol: MCH; multiwalled carbon nanotubes: MWCNTs; palladium nanoparticles: Pd NPs; poly(methylmetacrylate: PMMA; pyrrole-nitrilotriacetic acid monomer: PPY-NTA; platinum nanoparticles: PtNPs; reduced graphene oxide nanoribbon: rGONR.

### 4.3. Estrogen Receptor-Based Biosensors

Human-estrogen receptor alpha (ER-α) is a protein that belongs to the nuclear receptor group and can bind xenoestrogens such as 17β-estradiol. Due to its specificity and ability to be engineered, ER-α was used as bio-recognition element for the development of ED detection methods [86]. Estrogen receptor-based biosensors are not often encountered, but it is worth mentioning some notable works (Table 6).

Im et al. [88] developed an EIS biosensor for 17β-estradiol based on the biding of estrogen to the surface-immobilized estrogen. The surface modification of the Au electrode involved the use of 3-mercaptopropionic acid (3-MPA), which binds to the Au surface via thiol groups and the ECD-NHS chemistry for the covalent binding of the estrogen receptor-alpha (ER-α) to the carboxyl groups of 3-MPA. The hormone has been detected at a concentration of 10^−6^ M. The biosensor was developed further by the same group [89] by using BSA to block the remaining binding sites. The dynamic range was within 1 × 10^−13^–1 × 10^−9^ M with a LOD of 1 × 10^−13^ M.

### 4.4. Enzyme-Based Biosensors

Other biorecognition elements, such as enzymes, were used to develop the ED biosensors. In the case of phenolic compounds, these biosensors are often based on the enzymatic oxidation by enzymes such as tyrosinase [91] or laccase [92]. Metal composites have been used to modify the working electrodes and to provide a large surface area for enzyme immobilization and improved surface charge transfer.

**Figure 5:**
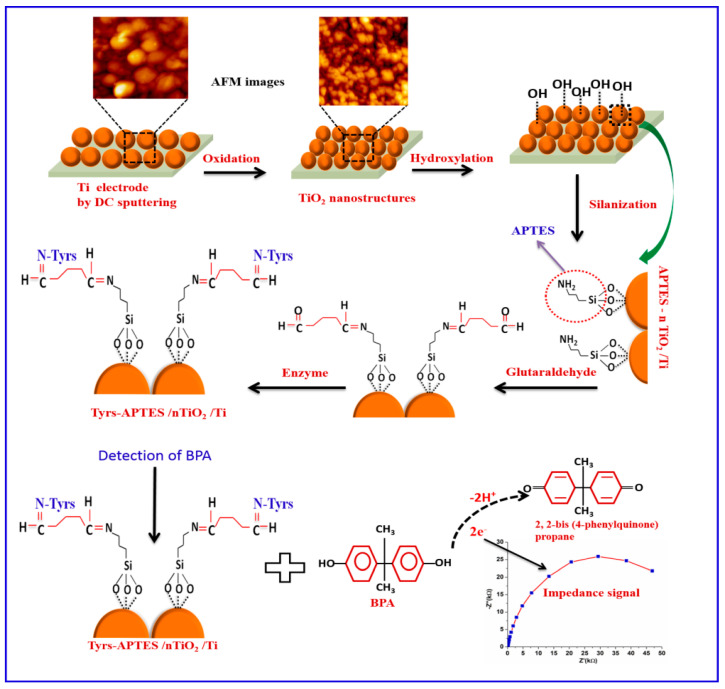
Preparation of the Tyrs– APTES /nTiO_2_/Ti biosensor: the Ti electrode was modified using self-assembled direct current (DC) sputtered nanostructured rutile TiO_2_ aiming the covalent immobilization of tyrosinase. The oxidation of BPA caused changes in the charge transfer properties of the interface, monitored through EIS measurements. Reproduced from [93] with permission of Elsevier.

Singh et al. [93] developed a tyrosinase biosensor for the impedimetric detection of BPA. The sensing surface used was based on nanostructured TiO_2_ that was functionalized further with an 3-Aminopropyltriethoxysilane (APTES)-based SAM and glutaraldehyde. The enzyme tyrosinase was covalently immobilized on the modified surface. The immobilized tyrosinase oxidized BPA to 2,2-bis(phenylquinone)propane and the resulting electrons were transferred to the nTiO_2_/Ti electrode, which led to a decrease in the R_ct_ value (Figure 5). A linear relationship between variation of the R_ct_ and concentration of BPA was observed within a linear range of 0.01–1.0 μM. The Tyrs–APTES/nTiO_2_/Ti biosensor achieved a LOD of 0.01 μM.

Recently, a laccase biosensor for BPA detection was developed using a conjugate containing reduced graphene oxide and ferrous-ferric oxide nanoparticles (rGO-Fe_3_O_4_ NPs) [94]. Chit95 (chitosan with a degree of deacetylation of 95%) was used for laccase immobilization. The modified biosensor was used for both amperometric and impedimetric detection. Laccase (source: *Trametes versicolor*) catalyzes the oxidation reaction of p-diphenols. The biosensor provided a linear range of 0.025–20 μM and a LOD of 65 nM using EIS detection. 

### 4.5. Peptide-Based Biosensors

Peptides are oligomers and polymers that can be customized with highly controlled preparation methods, due to the variety of natural and synthetic amino acids available for synthesis. Peptides have been employed in biosensing due to their specificity, better chemical and conformational stability compared to antibodies [95], low cost and facile synthesis and modification protocols that allow customization for a wide variety of applications [96].

Gutés et al. [97] reported a novel peptide-based biosensor for the detection of decabromodiphenyl ether (DBDE). The supporting electrode was prepared by growing graphene on a copper foil, decorating with AuNPs and spin-coating with poly(methylmetacrylate) (PMMA). The composite materials were transferred on a GCE. The DBDE binding peptide sequence, WHWNAWNWSSQQ, was immobilized by incubation of the AuNP-functionalized graphene electrode. The peptide-AuNP-graphene modified electrode was used for the EIS determination of polybrominated diphenyl ethers (PBDEs) and provided a response to molecules with similar structure and the biosensor showed little interference from similar compounds such as diphenyl ether.

### 4.6. Microbial Biosensors

Microbial biosensors use microorganisms as a sensitive biological element. Their main advantage is the fact that, unlike molecule-based biosensors, they provide information on toxicity or bioavailability [98]. The microbes are usually genetically engineered by modifying their structure to serve as bio-receptors for the target molecule [99].

Furst et al. have developed an electrochemical sandwich assay that measures the total estrogenic activity of a sample [100]. *E. coli* cells were engineered to display the estrogen receptor α (ER-α) capture agent, while a synthesized antibody mimic protein was immobilized on a gold electrode via cysteine gold chemistry. The ED molecules first bind the receptor proteins anchored onto the surface, then *E. coli* cells from solution attach to the receptor-bound ED, thus causing the increase in EIS signal. The system was used for the detection of estrogenic activity in solutions containing 17β-estradiol, 4-nonylphenol, genistein and DES. The calculated LOD for 17β-estradiol was 500 pM. The method did not detect individual compounds but allowed the estimation of the total estrogenic activity.

## 5. Conclusions

EIS (bio)sensors have been increasingly used over the past decade since they are versatile, easy to functionalize and amenable for on-site field detection. Novel materials and capture elements have been developed to enhance the performance of ED detection even in complex matrices. Still, in label-free assays, EIS has to be used with precaution for avoiding false positive results, related to the interface dynamics and its inherently low signal-to-noise ratio [101]. In the absence of a laborious experimental control associated with an understanding of EIS concepts, variations caused by drift or non-specific binding can be mistakenly interpreted as specific interaction, thus compromising the analysis outcome. Although Faradaic EIS platforms are overwhelmingly used for label free detection, more and more papers have reported EIS sensors with surface-confined redox probes, where the non-Faradaic methodology (i.e., measuring changes in the dielectric properties at the target binding) was better suited for cost-effective, ultra sensitive and at point-of-use devices [102]. Due to their tunable nature and simple design, MIPs, aptamer, and peptide-modified biosensors represent reliable, inexpensive alternatives to the detection methods that use classical capture elements. Biosensors with biomolecules immobilized on different functional nanomaterials, (carbon nanotubes, graphene, graphite and related) display an increased number of the binding sites, enhanced stability, and facile electron transfer. Aptasensors are among the most sensitive biosensors, allowing the detection of the femtomolar concentration of several EDs, such as BPA. The implementation of Faradaic and non-Faradaic sensors in microfluidic systems allows rapid detection with minimal consumption of samples and reagents. Additional efforts are required to develop performing “lab-on-a-chip” sensing devices by coupling EIS detection with mass-sensitive techniques such as surface acoustic wave (SAW) and surface plasmon resonance (SPR). These tandem-interrogation concepts may provide promising tools for the development of robust, user-friendly sample-to-answer platforms.

## Figures and Tables

**Table 2:** Impedimetric MIP-based sensors for EDs detection.

Analyte	Platform	Linear Range	LOD	Real Sample	Advantages	Limitations	Ref.
**E2**	MIP/GCE	1 aM–1 μM	0.36 aM	human serum	Low detection limit; High stability	Multiple preparation and optimization steps	[44]
**ATZ**	MIP/GFE	5–20 ppm	-	-	High selectivity; Simple modification protocol	Narrow linear range	[45]
**BPA**	E-MIP/ITO	1–12 mM	0.42 mM	-	Selectivity	Low sensitivity; High detection limit; Narrow linear range	[46]
**DBP**	MIP-PPY/PGE	0.01–1 μM	4.5 nM	-	Simple modification protocol	Relatively high detection limit	[47]
**DDT**	PDA@Fe_3_O_4_-MIP MNPs in solution, EIS measurements on GCE	1 × 10^−11^–1 × 10^−3^ M	6 × 10^−12^ M	radish	Reusability; Wide linear range	Long assay time; Multiple preparation and separation steps; Requires a magnet	[42]
**DEHP**	MIP-APTES SAM/AuIDE	10–100 ppm	-	-	Low sample volume	Narrow linear range; Requires electrode fabrication	[48]
**DEHP**	MIP/AuIDE, capacitive sensor	10–200 ppm	-	-	Simple modification protocol	Limited sensor reusability; Narrow linear range	[49]
**Testosterone**	poly(o-PD) MIP/GO/GCE	1 fM–1 μM	0.4 fM	human serum	Fast response time; Low detection limit; High stability	-	[50]
**Tributyltin**	MIP-Fe_3_O_4_NPs/SPE	5 pM–5 μM	5.37 pM	sea water	Large active surface area; High sensitivity; Wide linear range	Multiple separation and washing steps; Requires a magnet	[51]
**ZEN**	poly(o-PD) MIP/SPGE	2.5–200 ng/mL	2.5 ng/mL	corn flakes	High selectivity; Simple modification protocol; Short incubation time	Narrow linear range	[43]

3-Aminopropyltriethoxysilane: APTES; Au interdigitated electrode: AuIDE; gold nanoparticles: AuNP; electropolymerized molecularly imprinted polymer: E-MIP; graphite felt electrode: GFE; graphene oxide: GO; indium tin oxide: ITO; pencil graphite electrode: PGE; polypyrrole: PPY; self-assembled monolayer: SAM; screen-printed electrode: SPE; screen-printed graphene electrode: SPGE.

**Table 3:** Impedimetric sensors for EDs detection with graphene, carbon-nanotubes or cyclodextrins.

Analyte	Platform	Linear Range	LOD	Real Samples	Advantages	Limitations	Ref.
BPA	Fe(III)TMPP/TRGO/Au	1 × 10^−12^–1 × 10^−8^ M	2.1 × 10^−13^ M	fresh milk	High selectivity; Wide linear range	Additional preparation steps for the electrode materials	[56]
DEHP	β-CD–GO/ GCE	2–18 μM	0.12 μM	wastewater from plastics factory	High selectivity	Narrow linear range; Requires sample deoxygenation	[57]
DEHP	DEHP/β-CD/G/DAD/ GCE	0.2–1.2 μM	0.01 μM	river water	Good stability	Multiple preparation and optimization steps; Narrow linear range	[58]
PCB-77	PyCD/SWCNT/GCE	2–10 μM	1 nM	-	High selectivity	Long preconcentration time (3h); Narrow linear range	[55]

b-cyclodextrin: β-CD; 1,10-diaminodecane: DAD; graphene quantum dots: GQD; 3,3′,4,4′-tetrachlorobiphenyl: PCB-77; pyrenecyclodextrin: PyCD; single-walled carbon nanotube: SWCNT; thermally reduced graphene oxide: TRGO; triflato 5,10,15,20-tetrakis (4-metoxyphenyl) porphyrinato iron (III): Fe(III)TMPP.

**Table 6:** Estrogen receptor-based impedimetric biosensors for the detection of EDs.

Analyte	Platform	Linear Range	LOD	Real Samples	Advantages	Limitations	Ref.
**E2**	ER-α/AuNPs/s-BLM/Pt	5–150 ng/L	1 ng/L	river water	Does not require blocking of non-specific sites; Label-free; Simple modification protocol	Low stability; Narrow linear range; Only detects total estrogenic activity	[87]
**E2**	ER-α/3-MPA/Au	-	-	-	Label-free; Simple modification protocol	Only detects total estrogenic activity; Requires blocking of non-specific sites	[88]
**E2**	ER-α/3-MPA/Au	1 × 10^−13^–1 × 10^−9^ M	1 × 10^−13^ M	human urine	Label-free; Simple modification protocol; Wide linear range	Longer incubation time (90 min); Only detects total estrogenic activity; Requires blocking of non-specific sites	[89]
**E2**	ER-α/3-MPA/Au	3.7 × 10^−4^–3.7 ng/L	3.7 × 10^−4^ ng/L	-	Label-free; Simple modification protocol	Only detects total estrogenic activity; Requires blocking of non-specific sites	[90]

3-mercaptopropionic acid: 3-MPA; bilayer lipid membranes: s-BLM.

## Abbreviations

1-AP-CNHs, 1-aminopyrene modified carbon nanohorns; 3D GF, 3D graphene; 11-MUA, 11-mercaptoundecanoic acid; 16-PHA, 16-phosphonohexadecanoic acid; β-CD, β-cyclodextrin; β-MnO2 CNs, manganese oxide cacti-like nanostructures; AAP, acetamiprid; Ab, antibody; ACA, anti-cortisol antibody; ACEK, alternating current electro-kinetics; ACET, alternating current electrothermal; AOO, anodized aluminum oxide; APTES, 3-Aminopropyltriethoxysilane; Apt, aptamer; AuIDE, Au interdigitated electrode; AuNCs, Au nanocones; AuNP, gold nanoparticle; ATZ, atrazine; BDD, boron-doped diamond; BDE-47, 2,2′,4,4′-Tetrabromodiphenyl ether; C60, fullerene C60; CBZ, carbendazim; Cdl, double-layer capacitance; CDs, carbon dots; C-ELISA, competitive enzyme-linked immunosorbent assay; CNHs, carbon nanohorns; DAD, 1,10-diaminodecane; DBP, dibutyl phthalate; DBDE, decabromodiphenyl ether; DDT, Dichlorodiphenyltrichloroethane; DEHP, di(2-ethylhexyl) phthalate; E2, 17β-estradiol; EDC, N-(3-dimethylaminopropyl)-N-ethylcarbodiimide hydrochloride; EIS, electrochemical impedance spectroscopy; E-MIP, electropolymerized molecularly imprinted polymer; ER-α, estrogen receptor-alpha; ET, electron transfer; Fe(III)TMPP, triflato 5,10,15,20-tetrakis(4-metoxyphenyl)porphyrinato) iron(III); GCE, glassy carbon electrode; GFE, graphite felt electrode; GO, graphene oxide; GOPTS, (3-glycidyloxypropyl) triethoxysilan; GQD, graphene quantum dots; IDE, interdigitated electrode; ITO, indium tin oxide electrode; MB, methylene blue; MCH, 6-mercapto-1-hexanol; MC-LR, microcystin-LR; MEA, monoethanolamine; MPA, Mercaptopropionic acid; MIPs, molecularly-imprinted polymers; MNF, electrospun manganese oxide nanofibers; MNPs, magnetic nanoparticles; MOF, metal–organic framework; MWCNTs, multi-walled carbon nanotubes; nano-CP, nano-particle comprised conducting polymer; NHS, N-hydroxysuccinimide; NorFLX, norfluoxetine; o-PD, o-phenylenediamine; PBDEs, polybrominated diphenyl ethers; PCBs, polychlorinated biphenyls; PCB-77, 3,3′,4,4′-tetrachlorobiphenyl; PdNPs, palladium nanoparticles; PDA, polydopamine; PFOS, perfluorooctane sulfonate; PGE, pencil graphite electrode; poly(Gly), polyglycine; PMMA, poly(methylmetacrylate); PPY, polypyrrole; PPY-NTA, pyrrole-nitrilotriacetic acid monomer; Pt NPs, platinum nanoparticles; PyCD, pyrenecyclodextrin; R_c_t, charge-transfer resistance; Rmt, mass transfer resistance; rGO, reduced graphene oxide; rGONR, reduced graphene oxide nanoribbon; SAMs, self-assembled monolayer; SAW, surface acoustic wave; s-BLM, bilayer lipid membranes; SPA, 3-sulfanylpropionoic acid; SPCE, screen-printed carbon electrode; SPE, screen-printed electrode; SPGE, screen-printed gold electrode; SPR, surface plasmon resonance; SWCNT, single-walled carbon nanotube; W, Warburg impedance; ZEN, zearalenone; ZnONR, Zinc Oxide nanorods.
